# Association of Plasma Zinc and Copper with Body Composition, Lipids and Inflammation in a Cross-Sectional General Population Sample from Germany

**DOI:** 10.3390/nu15204460

**Published:** 2023-10-20

**Authors:** Cara Övermöhle, Gerald Rimbach, Sabina Waniek, Eike A. Strathmann, Tatjana Liedtke, Paula Stürmer, Marcus Both, Katharina S. Weber, Wolfgang Lieb

**Affiliations:** 1 Institute of Epidemiology, Kiel University, 24105 Kiel, Germanykatharina.weber@epi.uni-kiel.de (K.S.W.); wolfgang.lieb@epi.uni-kiel.de (W.L.); 2 Institute of Human Nutrition and Food Science, Kiel University, 24118 Kiel, Germany; 3 Department of Diagnostic Radiology, University Hospital Schleswig-Holstein, 24105 Kiel, Germany

**Keywords:** abdominal adiposity, magnet resonance imaging, popgen, trace elements

## Abstract

We aimed to relate circulating plasma zinc and copper to a broad spectrum of adiposity-related traits in a cross-sectional Northern German study (n = 841, 42% female, age: 61 ± 12 years). Zinc and copper were measured by inductively coupled plasma–mass spectrometry. Subcutaneous (SAT) and visceral (VAT) adipose tissue and liver fat were derived from 534 and 538 participants, respectively, via magnet resonance imaging. Associations were assessed using multivariable-adjusted linear regression analysis. An increase per one standard deviation (SD) in zinc was associated with direct linear increases in body mass index (BMI) (1.17%; 95% confidence interval (95%CI) 0.15–2.20%), waist circumference (0.85%; 95%CI 0.04–1.67%) and waist-to-hip ratio (0.64%; 95%CI 0.18–1.09%). A 1-SD increment in copper was directly associated with BMI (1.64%; 0.41–2.88%) and waist circumference (1.22%; 95%CI 0.25–2.20%) but not waist-to-hip ratio. Independent of fat intake, zinc displayed associations with VAT (5.73%; 95%CI 2.04–9.56%) and with liver fat (3.84%; 95%CI 1.49–6.25%), the latter association being also independent of BMI. Copper was directly associated with SAT (4.64%; 95%CI 0.31–9.15%) before accounting for BMI, but showed no association with VAT or liver fat. Observed associations suggest a possible relevance of zinc and copper to adiposity. Particularly zinc displayed associations with traits of abdominal adiposity and liver fat.

## 1. Introduction

Zinc (Zn) is an essential trace element for human health. Approximately 300 enzymes contain Zn. Thus, Zn is involved in a variety of biological processes, including growth, adequate immune response, protection against oxidative stress and aging [1,2]. Furthermore, Zn is involved in lipid storage and metabolism [3,4,5]. Pronounced Zn deficiency is rare in Europe (<5%) [6] and is clinically characterized by skin rash, impaired wound healing and susceptibility for infections [1,2].

A second essential trace element for human health is copper (Cu). Cu supports enzyme activity, iron metabolism, connective tissue formation and antioxidant defense [7,8,9]. Both a Cu deficiency and excess can have adverse health effects. While Cu deficiency is characterized by anemia, neurological impairments and impaired immune function [7,8], excess Cu may lead to liver damage, cognitive impairments and hemolysis [8,9].

In many biological processes, Zn and Cu act antagonistically, since they compete for absorption and metallothionein binding [10]. Evaluating both trace minerals together is therefore essential to fully understand their function in the human body.

Mounting evidence links Zn and Cu to adiposity. A variety of studies have reported associations of these trace minerals with different measures of body fat and body composition. In a Korean study (n = 1896), higher serum Zn was directly associated with increased waist circumference (WC) and body fat (measured by dual-energy X-ray absorptiometry (DEXA)) in men but not in women [11]. Similar associations were found for serum Cu in studies in US adults (n = 7285) [12] and in Chinese children (n = 454) [13].

However, results from previous studies have been conflicting. A study in Mexican adults (n = 172) reported inverse associations between serum Zn and body mass index (BMI) [14], while no differences in serum Zn and Cu were observed between children from Montenegro with normal weight and obesity (n = 202) [15].

Experimental studies corroborate the association of Zn with lipid storage and metabolism [5]. In mice models, chronic Zn supplementation led to more visceral adipose tissue and an increased adipocyte size [4]. In rat hepatocytes, treatment with high levels of Zn, led to an upregulation of different enzymes involved in lipid metabolism, including fatty acid synthase, stearoyl-CoA desaturase-1, and acetyl-CoA carboxylase 1 [3].

As mentioned above, previous studies used DEXA to determine associations of Zn and Cu with body composition. However, it is known that magnet resonance imaging (MRI) is considered even more precise to determine body composition [16].

Therefore, we aimed to investigate in a community-based sample from Northern Germany whether (i) plasma Zn and Cu concentrations were related to a broad spectrum of anthropometric, lipid and inflammatory markers; (ii) both trace elements were associated with MRI traits of body composition; and (iii) the associations of both trace elements with these traits were in opposite directionality.

## 2. Subjects and Methods

### 2.1. Study Sample

**Figure 1:**
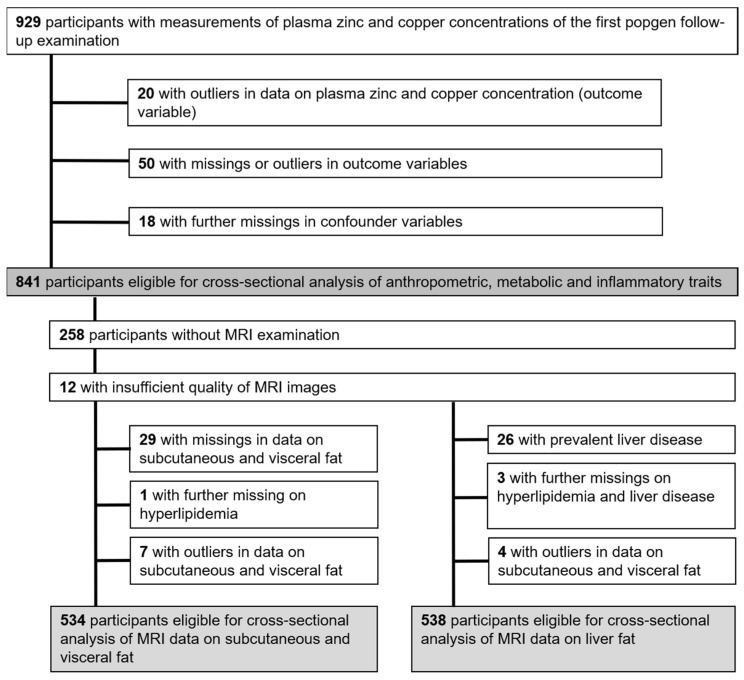
Flowchart showing the participants from the first follow-up examination (2010–2012) in the popgen cohort from Northern Germany being eligible for analysis from those with measurements of plasma zinc concentrations. Abbreviations: MRI, magnet resonance imaging.

The data for this cross-sectional study were obtained from the first follow-up examination (2010–2012) of the “popgen controls”, a prospective population-based cohort in Northern Germany. Initially, 1317 participants were enrolled through population registries (n = 747) and blood donor registries from the University Hospital in Kiel (n = 570). Out of these, 929 participants attended the first follow-up examination [17,18,19]. Data on Zn and Cu biomarkers, outcomes, and relevant confounders were analyzed in 841 participants (Figure 1). MRI data for subcutaneous and visceral adipose tissue, along with liver fat content, were analyzed in a subset of 534 (adipose tissue) and 538 (liver fat content) individuals, respectively.

The study adhered to the Declaration of Helsinki, and the Ethics Committee of the Medical Faculty of Kiel University approved the study (Project identification code A 156/03; P2N reference numbers 2021-036, 2022-048). All participants provided written informed consent.

### 2.2. Clinical Evaluation and Laboratory Analyses

Trained personnel conducted standardized physical examinations [18,19,20]. Self-administered questionnaires collected data on age, sex, education, medical history, and lifestyle factors, including smoking habits and habitual physical activity over the past 12 months. Total physical activity was calculated by summing up the metabolic equivalent of the task for each activity [18,19,20,21].

Weight and height were measured with participants wearing light clothing [18]. An amount of 2.0 kg was subtracted from the measured weight to correct for clothing. Based on these measurements, BMI (kg/m^2^) was calculated. Additionally, WC and hip circumference were measured to calculate the waist-to-hip ratio (WHR).

Laboratory analyses, including assessment of triglycerides (TG), high-density lipoprotein (HDL) and low-density lipoprotein (LDL) cholesterol and plasma C-reactive protein (CRP), were performed using the unfrozen samples the same day in the Institute of Clinical Chemistry at the University Hospital Schleswig-Holstein, Campus Kiel [18,19,20].

#### 2.2.1. Assessment of Plasma Zinc and Copper

Concentrations of Zn and Cu were measured from frozen plasma samples in 2022 by SGS Analytics Germany GmbH, via inductively coupled plasma–mass spectrometry ICAP Q instrument (Thermo Fisher Scientific, Waltham, MA, USA) in accordance with DIN EN ISO 17294-2: 2017-01 [22]. Prior to measurement, plasma sample aliquots were decomposed (nitric acid and hydrogen peroxide (4:1)) by microwave digestion [23]. Multi-element standard was used for calibration, while rhodium (2 µg/L) was added as internal standard. Spectroscopic interferences were minimized by use of a collision/reaction cell for detection. 

#### 2.2.2. Assessment of Dietary Intake of Zinc, Total Fat and Saturated Fatty Acids

Dietary intake of Zn, Cu, total fat and saturated fatty acids (SFAs) was approximated by a food frequency questionnaire (FFQ). The FFQ has been validated for the German population within the EPIC-Potsdam study [24]. Based on the FFQ data, nutrient content was calculated according to the German Food Code and Nutrient Database (version II.3) [24,25].

#### 2.2.3. Assessment of Subcutaneous and Visceral Adipose Tissue and Liver Fat

MRI measurements of subcutaneous (SAT) and visceral (VAT) adipose tissue and liver fat were conducted using a 1.5 T scanner (Magnetom Avanto; Siemens Medical solution, Erlangen, Germany) [26]. Details of the procedure have been described elsewhere [20,27,28]. Adipose tissue volumes (dm^3^) were calculated by the number of voxels multiplied by the voxel size [26,27]. For SAT, the voxels beneath the skin, surrounding the abdomen, from the top of the liver to the femoral heads, was determined. VAT was defined as voxels counted from the top of the liver to the femoral heads inside the abdominal muscular wall. Liver signal intensity (LSI) was used to determine liver fat by computing the relative difference of LSI on out-of-phase and in-phase images in arbitrary units [20,26]. Images of both phases were captured during breath hold using T1-wheighted gradient echo sequences. LSI was determined by measurement of the average of three circular regions of the liver parenchyma.

### 2.3. Definitions

Fatty liver disease was defined as ln-transformed LSI ≥ 3.0 [20,26]. The education of participants was given in three categories: low (≤9 years), middle (10 years), or high (≥11 years) [18,23]. Smoking behavior was presented as three groups: never-smokers, former smokers and current smokers [18,23,26]. Fasting status at time of blood sampling was defined as not fasting (ate or drank within the last 8 h) and fasting (did not eat or drink in the last 8 h). The season in which the examination took place was presented in the following categories: winter (January–March), spring (April–June), summer (July–September) and fall (October–December) [23].

### 2.4. Statistical Analyses

Data analyses were performed using SAS^®^ (version 9.4; SAS Institute, Cary, NC, USA) and *p* values of <0.05 were considered statistically significant.

Only participants with complete and plausible data for independent, dependent and confounding variables were included. Participants with outliers in continuous independent or dependent variables were removed from the sample. Fences for outliers were defined as mean ± 3× standard deviation (SD). Potential confounders were defined a priori based on the literature.

In descriptive analysis, categorical variables were reported as absolute numbers and percentages, continuous normally distributed variables as mean and SD, and continuous skewed variables as median and interquartile range. Sample characteristics were compared across tertiles of plasma Zn concentrations (µg/L) using a chi-square test for categorical variables, unadjusted general linear models for continuous normally distributed variables and the Kruskal–Wallis test for continuous skewed variables [23].

#### 2.4.1. Association of Zinc and Copper with Anthropometric, Metabolic and Inflammatory Parameters

Linear regression analyses (PROC GLM, SAS^®^) were used to assess the associations of Zn and Cu (each trace element considered as a separate exposure variable) with anthropometric parameters (BMI, WC, and WHR), lipid traits (TG, HDL, and LDL) and inflammation (CRP) (separate dependent outcome variables, all ln-transformed). We performed the following adjustments: Model A1 was unadjusted. Model A2 was adjusted for age and sex. Model A3 additionally included BMI (not for WC and WHR), education, smoking habits, season, physical activity, alcohol consumption, Zn supplementation (for Zn as an independent variable), lipid-lowering medication (only for lipid traits) and fasting status (only for lipid traits). Model A4 was additionally adjusted for total fat intake and intake of SAFs.

The effect estimates from the linear regression models were re-transformed and given as % increase in the dependent variable per 1-SD increase in the independent variable (Zn or Cu).

#### 2.4.2. Association of Zinc and Copper with MRI Traits of Body Composition

To model the associations of Zn and Cu (as separate exposure variables) with ln-transformed MRI traits of body composition (SAT, VAT and LSI, each trait considered as a separate outcome) we used multivariable-adjusted linear regression models (PROC GLM, SAS^®^) with the following adjustments: Model B1 was unadjusted. Model B2 was adjusted for age and sex. Model B3 was additionally adjusted for education, smoking habits, season, physical activity, alcohol consumption, Zn supplementation (for Zn as independent variable), lipid-lowering medication and prevalent fatty liver disease (for LSI as the outcome variable. Model B4 additionally included total fat intake and intake of SAFs, while Model B5 also included BMI.

The effect estimates from the linear regression models were re-transformed and given as % increase in the dependent variable per 1-SD increase in the independent variable (Zn or Cu).

#### 2.4.3. Sensitivity Analysis

In order to assess if the observed results might have been affected by use of Zn supplementation, all analyses as described above were repeated in a subsample of participants not taking Zn supplements (n = 784) and in the corresponding MRI subsamples for adipose tissues (n = 493) and liver fat (n = 497).

## 3. Results

### 3.1. Participants’ Characteristics

Baseline characteristics of our study sample are provided in Table 1. About 7% of the participants reported intake of Zn supplements. According to a previous classification [29], only 3.8% (n = 32) met the criterion for Zn deficiency (<460 µg/L), while 1.9% (n = 16) had low (<760 µg/L in females, <700 µg/L in males) and 2.1% (n = 18) high (>1522 µg/L in females, >1400 µg/L in males) plasma concentrations for Cu.

Across Zn tertiles, comparable plasma Cu and FFQ extracted intakes of dietary Zn, Cu, fat and SAFs were established (Table 1). Participants in the top Zn tertile had higher concentrations of plasma LDL as compared to the lower tertiles. Additionally, alcohol intake was highest in the lowest tertile of Zn concentrations.

Due to missing and implausible data, not all participants were included in our main analyses. Compared to the excluded participants (n = 88), participants within our initial study sample (n = 841) had comparable circulating Zn concentrations, lower circulating Cu concentrations, were older, had lower BMI, were less likely smokers and had higher physical activity (Table S1).

### 3.2. Association of Zinc and Copper with Anthropometric, Metabolic and Inflammatory Traits

In multivariable-adjusted linear regression models, Zn was directly associated with different anthropometric measures (i.e., BMI, WC and WHR) and LDL, and inversely associated with CRP (Table 2, Model A3, *p* = 0.023; 0.041; 0.007; <0.001 and 0.007, respectively). Cu displayed direct associations with BMI, WC, HDL, LDL, and CRP (Table 2, Model A3, *p* = 0.008; 0.015; 0.002; 0.010 and <0.001, respectively). Including intake of total fat and SAFs into the model did not change results considerably (Table 2, Model A4).

Restricting the sample to participants without intake of Zn supplements led to essentially unchanged results (Table S2).

### 3.3. Associations of Zinc and Copper with Adipose Tissue and Liver Fat

Zn displayed direct associations with SAT, VAT and LSI in multivariable-adjusted model B3 (Table 3, Model B3, *p* = 0.049; 0.002 and 0.001, respectively). Further adjustment for total fat intake and intake of SAFs attenuated the association with SAT (Table 3, Model B4, *p* = 0.065). Upon further adjustment for BMI, the association with LSI remained statistically significant (Table 3, Model B5, *p* = 0.006), but the associations with VAT were rendered statistically non-significant (Table 3, Model B5, *p* = 0.057).

Cu was directly associated with SAT upon adjustment for fat and SAFs intake (Table 3, Models B3 and B4, *p* = 0.031 and 0.036). However, additional adjustment for BMI rendered this association statistically non-significant (Table 3, Model B5, *p* = 0.688). VAT and LSI were not related to plasma Cu levels (Table 3).

In sensitivity analysis restricted to participants not taking Zn supplements, associations of Zn and Cu with SAT, VAT and LSI remained essentially unchanged. However, exclusion of participants taking Zn supplements led to a more pronounced association of Zn with VAT, which now remained statistically significant upon further adjustment for BMI (Table S3). Cu was no longer associated with SAT in this subsample (Table S3).

MRI examinations were conducted in a subsample. Compared to the excluded participants, participants in the MRI subsample had lower Cu blood concentrations, were slightly older and less likely smokers, had lower BMI, drank more alcohol and had higher education (Table S1).

Restricting the sample to participants without intake of Zn supplements led to essentially unchanged results (Table S2).

## 4. Discussion

In a moderate-sized sample from Northern Germany, we evaluated the associations of the trace elements Zn and Cu with a broad spectrum of anthropometric, metabolic and inflammatory markers, and with MRI traits of body composition (SAT, VAT, and liver fat).

Our principal observations were as follows: first, Zn and Cu both displayed direct associations with anthropometric and lipid traits. Second, Zn displayed direct associations with MRI traits of body composition, including SAT, VAT and liver fat in multivariable-adjusted models excluding BMI. However, upon additional adjustment for BMI, only the association with liver fat remained statistically significant in the overall sample. In a sample excluding participants on Zn supplements, both, liver fat and VAT remained statistically significantly associated with Zn concentrations in multivariable-adjusted models including BMI. Third, Cu levels were not related to SAT, VAT and liver fat in multivariable-adjusted models including BMI. Fourth, intake of total fat or SAFs could not explain the associations between Zn and Cu, and features of lipid metabolism and fat storage. Fifth, Zn and Cu showed an opposing effect on CRP, while all other associations were consistently in the same direction.

### 4.1. Associations of Zinc and Copper with Anthropometric Traits and MRI Measures of Body Composition

Prior analyses on the association of Zn and Cu with anthropometric traits were not entirely consistent and used different measures of Zn and Cu exposure, including dietary intake and circulating Zn and Cu concentrations.

In 19952 participants from the US National Health and Nutrition Examination Survey (NHANES) (2007–2014), dietary Zn intake was inversely associated with BMI and WC [30]. Additionally, in a recent meta-analysis, serum Zn concentrations were inversely related to BMI [31]. In our sample, however, plasma levels of both Zn and Cu were directly associated with BMI and WHR in multivariable-adjusted models, which is in part in line with results from a large observational survey from Korea (NHANES, 2010) (n = 1896), where higher serum Zn was associated with larger WC in male participants, but not in females [11].

Also, the associations of Cu with body composition and body weight are in part conflicting [12,30,32,33]. A meta-analysis from 2019 reported an association of higher Cu concentrations and overweight [34], which is consistent with our observations.

We expand these prior analyses by relating plasma Zn and Cu levels to MRI traits of body composition. In these analyses, we observed direct linear associations of circulating Zn levels with SAT, VAT and liver fat in multivariable-adjusted models. Upon additional adjustment for BMI, only the association with liver fat remained statistically significant in the overall sample. However, in a sample excluding 57 individuals on Zn supplements, liver fat and VAT remained statistically significantly associated with Zn in multivariable-adjusted models including BMI. Cu concentrations were not consistently associated with VAT, SAT or liver fat. Some smaller studies on the association of these trait elements with MRI measures of obesity or with DEXA traits of body composition have been published. In 87 hemodialysis patients, a direct association of circulating Zn with VAT has been reported [35]. This agrees well with our observation in a much larger population-based sample. Furthermore, within the above mentioned Korean NHANES (n = 1896), a direct association of Zn with the quantity and distribution of body fat (assessed by DEXA) and abdominal obesity was found in in men (but not in women) [11]. Other studies, however, reported inverse associations between Zn levels and body fat % (assessed by DEXA) [36] or found no association of Zn concentrations with excess fat as measured by DEXA [15].

The association of Zn with adiposity is also supported by experimental studies and in animal models [3,5,37,38]. For example, Zn supplementation (200 mg/kg) for six weeks increased body fat by 49.4% and 18.9% in genetically obese mice and high-fat diet-induced obese mice, suggesting that body fat deposition can be aggravated by Zn supplementation [38]. In this context it has been hypothesized that Zn exerts an insulin-like effect or that it interacts with thyroid hormone activities [38]. In general, insulin suppresses lipolysis in adipocytes, leading to the accumulation of fat depots [39]. Therefore, insulin-like effects of Zn may also suppress lipolysis. Another explanation might be the regulation of Zn transporters within adipocytes, which differs between lean and obese subjects, and also between SAT and VAT, suggesting that metabolic activity in adipocytes may be dependent upon Zn and Zn transporters or vice versa [40]. Furthermore, the upregulation of lipogenic enzymes may be responsible for the observed associations in our sample [3,4,5]. For example, supplementation of Zn for 40 days increased the expression of fatty acid synthase, stearoyl-CoA desaturase-1, acetyl-CoA carboxylase 1, peroxisome proliferator-activated receptor gamma, and sterol-regulatory element binding protein in a study in piglets compared to a control diet without Zn supplementation [5].

We also observed a direct association of Zn with MRI-determined liver fat, further supporting an adipogenic role of Zn. This contrasts, however, with a recent meta-analysis reporting lower Zn concentrations in patients with non-alcoholic fatty liver disease (NAFLD) [41]. Nevertheless, the expression of lipogenic enzymes was shown to be upregulated in an in vitro study on rat hepatocytes [4], supporting our observed association of circulating Zn with increased liver fat.

Initial associations between Cu and SAT were weakened after further adjustment with BMI, while no associations emerged with VAT or liver fat (as measured by LSI). Also, the evidence regarding Cu’s influence on adipokines is partially conflicting [12]. NHANES data (2011–2016) from the USA (n = 7285) reported linear associations of Cu with anthropometric measures (BMI, and WC) and with adipose tissue (assessed by DEXA) [12]. Similarly, in children (n = 454), Cu was associated with increased body fat (measured by DEXA), partly mediated through inflammation [13]. In the US, NHANES 2011–2014 (n = 3211) reported higher Cu to be associated with an increased likelihood for NAFLD [9]. By contrast, a 2017 review suggests that reduced Cu availability might contribute to the development of NAFLD [42]. In our analyses, we observed no statistically significant relation of Cu with liver fat.

### 4.2. Association of Zinc and Copper with Blood Lipids

Both, Zn and Cu have been studied in relation to blood lipids in experimental, interventional and observational studies, in part with controversial results.

In a large Chinese observational study (n = 12028), higher Zn intake was associated with higher TG levels [43]. Another study by Kim and Choi (2013) reported no association of Zn intake with blood lipids in healthy adults with self-selected diet (n = 258) [44]. In 600 African Americans, Zn and also Cu were not associated with LDL, HDL or TG [45]. With respect to Cu, another cohort study from China reported that urinary Cu was directly associated with TG, but not with other blood lipids (n = 4812) [46].

It is noteworthy to mention that using dietary Zn intake as a measure of Zn status may be problematic because circulating Zn concentrations are tightly homeostatically regulated and, therefore, dietary intake of Zn may be a poor predictor of blood Zn levels [47]. In line with this concept, we observed no differences in dietary Cu and Zn intake across tertiles of plasma Zn in our sample.

Also, the results from the interventional studies and meta-analysis were partly conflicting. An intervention (n = 387) using 15 mg/day or 30 mg/day Zn supplement over 6 months raised LDL in middle-aged individuals taking 30 mg, but not in elderly or in the 15 mg group [48]. In contrast, an interventional study on Zn supplementation (50 mg Zn gluconate) in type 2 diabetes patients found reduced TG levels after 8 weeks [49].

It has to be acknowledged, however, that the daily intake of 30 mg or 50 mg per day is much higher than the average daily intake in our sample.

A 2015 meta-analysis of 24 studies with a total of n = 14515 participants supports Zn’s lipid-lowering effects [50].

On a parallel note, a meta-analysis of five randomized clinical trials (a total of n = 176 participants) reported no effect of Cu supplementation on blood lipids (TG, HDL and LDL) [51].

### 4.3. Diet

Dietary exposures are unlikely to play a significant role in observed associations of plasma Zn with blood lipids and body composition. Zn is mainly contained in meat, eggs and milk products, which are rich in fat and SAFs [52]. While Cu is also prevalent in fatty foods like liver, fish, nuts and chocolate, it is also contained in grain products [52]. In our analyses, the associations of Zn and Cu with anthropometric traits and MRI traits of body composition were not attenuated after adjustments for fat and SAFs, even though both nutrients have a strong influence on lipid metabolism and fat deposition themselves [53].

However, total fat and SAF intake were estimated by FFQ data. The concept behind the FFQ approach is to capture an individual’s dietary habits over an extended period, such as weeks, months, or even years, by assessing the habitual intake of a certain number of food items. Therefore, the FFQ-derived intake of specific single nutrients is rather a rough proxy for the actual intake. For macronutrients, the FFQ is an acceptable approximation of actual intake but, for subgroups such as SAFs and micronutrients, the agreement is rather weak [54,55]. It is possible that the use of FFQ data is not sufficient to completely rule out the possibility that the real driver behind the associations of obesity and body fat is fat intake.

### 4.4. Association of Zinc and Copper with Inflammation

While we observed directionality-consistent associations of Zn and Cu with anthropometric traits and lipids (see above), we observed opposing associations of the two trace elements—a direct association of Cu and an inverse association of Zn—with CRP, a marker of chronic systemic inflammation. The association of CRP with Cu is in line with previous reports and may be promoted by the upregulation of ceruloplasmin, the major carrier for Cu [56,57]. It has been furthermore suggested that inflammation may mediate the association of Cu with body composition.

A potential explanation for the inverse association of Zn with CRP could be that Zn levels may be lower in an inflammatory state, since Zn is needed to induce intracellular antioxidant pathways [57,58] in response to inflammation or that Zn levels are lower due to the downregulation of albumin, the carrier protein for Zn, in states of inflammation [56,57].

This opposing behavior of Zn and Cu may be due to an opposing reaction to certain inflammatory stimuli. For example, the cytokine interleukin 1 is produced under inflammatory conditions. On the one hand, interleukin 1 alters Zn distribution and thereby decreases circulating Zn levels and, on the other hand, the same cytokine increases ceruloplasmin, the major carrier for circulating Cu [59]. This mechanism illustrates why Zn and Cu behave differently under inflammatory conditions.

### 4.5. Strengths and Limitations

The strengths of our present study include the moderate-sized general-population sample and the availability of detailed information on multiple relevant confounders. Furthermore, MRI data were available for over 60% of participants, which enabled us to investigate traits of adipose tissue and liver fat. According to current knowledge, MRI is the most reliable method to analyze the body composition of humans [16]. The relatively large MRI subsample is therefore particularly valuable. Furthermore, plasma Zn is considered a reliable biomarker for Zn status in humans [60]. Evidence for plasma Cu as a reliable biomarker is not as clear [61]; however, it is used in large studies like NHANES from the US [9].

The following limitations warrant consideration. First, our cross-sectional design and the strongly pronounced multifunctionality of both trace elements does not allow causal inferences. Second, measuring dietary Zn, Cu, fat and SAF intake by FFQ is only a rough proxy for actual intake and the values should therefore only be viewed and evaluated with caution. Third, there were differences between included and excluded subjects, both in the large study sample and in the subsample for the MRI analysis. Overall, the included subjects were leaner than the excluded subjects and individuals with pronounced adiposity were not included in the present sample. It is known that severe adiposity is accompanied by chronic inflammation, which in consequence would lead to a decrease in Zn [62]. Thus, in a sample comprised by a large number of participants with severe adiposity, the observations we made could be masked. Fourth, our data were obtained in an elderly sample from Northern Germany. The generalizability of our observations to other age groups, ethnicities or geographic areas is unknown.

## 5. Conclusions

In our population-based sample from Northern Germany, plasma concentrations of Zn displayed a direct association with anthropometric traits (BMI, WC, and WHR) and MRI traits of body composition, i.e., SAT, VAT and liver fat. Cu displayed a direct association only with SAT, suggesting links of Cu and Zn to adiposity and body composition. Furthermore, we observed an association of Zn and Cu with LDL suggesting that both trace elements might be involved in lipid homeostasis. Finally, both trace elements were related with CRP, although with opposite directionality, indicating opposing roles of Zn and Cn in chronic systemic inflammation. The underlying physiological mechanisms need to be identified.

## Figures and Tables

**Table 1:** General characteristics of participants from the first follow-up examination (2010–2012) in the popgen cohort from Northern Germany according to sex-specific tertiles of circulating plasma zinc concentrations (n = 841).

	Total	T1	T2	T3	*p* Values ^d^
	434.9–958.7 µg/L	434.9–658.2 µg/L	658.3–724.9 µg/L	725.0–958.7 µg/L	
n (% female) ^a^	841 (42)	280 (41)	281 (44)	280 (40)	0.501
Age (years) ^b^	61 ± 12	60 ± 13	62 ± 12	61 ± 12	0.103
Plasma zinc (µg/L) ^b^	695.0 ± 83.1	607.7 ± 40.7	690.9 ± 18.9	786.3 ± 52.4	<0.001
Plasma copper (µg/L) ^b^	1037.9 ± 192.5	1035.9 ± 197.1	1043.9 ± 183.5	1034.0 ± 197.2	0.813
Zinc intake (mg/day) ^c^	11.1 (9.4; 13.2)	11.2 (9.3; 13.4)	11.1 (9.7; 13.0)	11.0 (9.3; 13.2)	0.865
Intake of zinc supplements (yes, (%)) ^a^	57 (7)	15 (5)	20 (7)	22 (8)	0.481
Copper intake (mg/day) ^c^	2.5 (2.1; 2.9)	2.5 (2.1; 3.0)	2.4 (2.1; 2.9)	2.4 (2.1; 2.8)	0.370
Intake of total fat (g/day) ^c^	94.1 (78.5; 115.9)	95.6 (77.7; 119.7)	94.1 (78.2; 112.8)	93.2 (79.1; 112.5)	0.783
Intake of saturated fatty acids (g/day) ^c^	38.0 (30.8; 46.9)	38.4 (29.8; 48.7)	38.1 (31.6; 45.5)	37.6 (30.6; 46.4)	0.838
Subcutaneous adipose tissue (dm^3^) (n = 534) ^c^	6.0 (4.7; 8.2)	5.9 (4.8; 8.1)	6.1 (4.5; 8.3)	6.1 (4.8; 8.2)	0.732
Visceral adipose tissue (dm^3^) (n = 538) ^c^	3.8 (2.4; 5.2)	3.5 (2.2; 5.1)	3.8 (2.5; 5.0)	4.1 (2.7; 5.2)	0.179
Liver signal intensity ^c^	18.5 (14.9; 23.5)	17.5 (14.5; 22.1)	19.0 (14.9; 23.8)	18.8 (15.3; 24.4)	0.067
Body mass index (kg/m^2^) ^b^	27.2 ± 4.2	27.0 ± 4.3	27.3 ± 4.2	27.4 ± 4.2	0.484
Waist circumference (cm) ^b^					
Females	90.3 ± 12.8	88.5 ± 12.4	91.5 ± 12.8	90.8 ± 13.2	0.172
Males	100.4 ± 10.8	100.6 ± 11.0	100.3 ± 10.9	100.4 ± 10.7	0.998
Waist-to-hip ratio ^b^					
Females	0.87 ± 0.07	0.86 ± 0.07	0.88 ± 0.06	0.88 ± 0.07	0.151
Males	0.98 ± 0.06	0.98 ± 0.06	0.99 ± 0.06	0.98 ± 0.07	0.661
Plasma triglyceride concentration (mg/dL) ^c^	105.0 (76.0; 138.0)	101.5 (77.5; 134.5)	103.0 (76.0; 131.0)	110.0 (73.0; 149.5)	0.301
Plasma high-density lipoprotein cholesterol (mg/dL) ^b^	65.1 ± 17.2	66.2 ± 17.7	64.2 ± 17.2	64.8 ± 16.8	0.397
Plasma low-density lipoprotein cholesterol (mg/dL) ^b^	131.6 ± 33.0	125.6 ± 34.2	133.8 ± 30.7	135.6 ± 33.4	<0.001
Lipid-lowering medication (yes, (%)) ^a^	114 (14)	30 (11)	36 (13)	48 (17)	0.077
C-reactive protein (mg/L) ^c^	1.2 (0.5; 2.4)	1.3 (0.5; 2.8)	1.2 (0.5; 2.4)	1.1 (0.5; 2.1)	0.203
Current smokers (yes, (%)) ^a^	108 (13)	44 (16)	32 (11)	32 (11)	0.263
Alcohol intake (g/day) ^c^	9.0 (3.2; 18.5)	10.7 (3.9; 21.9)	8.5 (3.0; 17.6)	8.4 (2.8; 16.4)	0.022
Physical activity (MET-hours/week) ^c^	90.5 (59.0; 131.5)	90.5 (59.7; 132.3)	88.3 (56.9; 126.3)	93.8 (59.1; 135.6)	0.694
Education level (low [<10 years], medium [10 years], high [≥11 years]) ^a^	292 (35); 271 (32); 278 (33)	87 (31); 93 (33); 100 (36)	106 (38); 90 (32); 85 (30)	99 (35); 88 (31); 93 (33)	0.517

Values are as follows: ^a^ n (%) (categorical variables), ^b^ mean ± SD (continuous normally distributed variables), ^c^ median (IQR) (continuous skewed variables), ^d^ *p* values based on chi-square test (categorical variables), Kruskal–Wallis test (continuous skewed variables) or general linear models (continuous normally distributed variables). Abbreviations: IQR, interquartile range; MET, metabolic equivalent of task; SD, standard deviation; T, tertile.

**Table 2:** Associations of plasma zinc and copper concentrations with anthropometric, metabolic and inflammatory traits (n = 841).

Anthropometric, Metabolic and Inflammatory Outcome Variables	Zinc		Copper	
	Estimates (95% CI) ^a^	*p* Values	Estimates (95% CI) ^a^	*p* Values
Body mass index (kg/m^2^)				
Model A1	1.35 (0.30; 2.40)	0.011	0.38 (−0.66; 1.42)	0.475
Model A2	1.19 (0.16; 2.23)	0.024	1.60 (0.38; 2.84)	0.010
Model A3	1.18 (0.17; 2.21)	0.023	1.65 (0.43; 2.89)	0.008
Model A4	1.17 (0.15; 2.20)	0.024	1.64 (0.41; 2.88)	0.009
Waist circumference (cm)				
Model A1	1.15 (0.23; 2.07)	0.014	−1.79 (−2.67; −0.89)	<0.001
Model A2	0.83 (0.02; 1.65)	0.046	1.26 (0.30; 2.24)	0.011
Model A3	0.84 (0.03; 1.66)	0.041	1.20 (0.23; 2.18)	0.015
Model A4	0.85 (0.04; 1.67)	0.040	1.22 (0.25; 2.20)	0.014
Waist-to-hip ratio				
Model A1	0.88 (0.25; 1.51)	0.006	−2.45 (−3.03; −1.85)	<0.001
Model A2	0.58 (0.12; 1.04)	0.014	0.67 (0.13; 1.22)	0.016
Model A3	0.63 (0.18; 1.09)	0.007	0.47 (−0.07; 1.02)	0.090
Model A4	0.64 (0.18; 1.09)	0.006	0.48 (−0.06; 1.03)	0.084
Plasma triglyceride concentration (mg/dL)				
Model A1	2.79 (−0.33; 6.00)	0.080	−0.89 (−3.90; 2.21)	0.569
Model A2	2.16 (−0.87; 5.29)	0.164	2.24 (−1.34; 5.97)	0.223
Model A3	1.58 (−1.33; 4.57)	0.290	−0.08 (−3.41; 3.36)	0.963
Model A4	1.52 (−1.39; 4.51)	0.310	−0.18 (−3.51; 3.26)	0.917
Plasma HDL concentration (mg/dL)				
Model A1	−1.58 (−3.33; 0.20)	0.082	7.49 (5.64; 9.37)	<0.001
Model A2	−1.26 (−2.85; 0.35)	0.124	1.40 (−0.53; 3.37)	0.156
Model A3	−0.50 (−1.99; 1.02)	0.517	2.85 (1.06; 4.67)	0.002
Model A4	−0.50 (−1.99; 1.03)	0.520	2.87 (1.07; 4.69)	0.002
Plasma LDL concentration (mg/dL)				
Model A1	3.32 (1.48; 5.18)	<0.001	3.17 (1.33; 5.03)	<0.001
Model A2	3.16 (1.34; 5.02)	<0.001	3.62 (1.46; 5.84)	0.001
Model A3	3.77 (1.99; 5.59)	<0.001	2.73 (0.66; 4.84)	0.010
Model A4	3.75 (1.97; 5.57)	<0.001	2.70 (0.63; 4.81)	0.010
C-reactive protein (mg/L)				
Model A1	−6.12 (−12.05; 0.20)	0.058	38.98 (30.70; 47.78)	<0.001
Model A2	−6.59 (−12.42; −0.36)	0.039	47.09 (36.86; 58.08)	<0.001
Model A3	−7.75 (−13.02; −2.17)	0.007	37.34 (28.41; 46.90)	<0.001
Model A4	−7.81 (−13.08; −2.22)	0.007	37.33 (28.38; 46.91)	<0.001

Model A1 was unadjusted. Model A2 was adjusted for age and sex. Model A3 was additionally adjusted for BMI (not for BMI, waist circumference and waist-to-hip ratio as exposure variable), education, smoking habits, season, zinc supplementation (for plasma zinc as dependent variable), lipid-lowering medication (for triglycerides, HDL and LDL as independent variables), fasting status (for triglycerides, HDL and LDL as independent variables) and physical activity and alcohol consumption. Model A4 was additionally adjusted for total fat intake and intake of saturated fatty acids. ^a^ Regression coefficients indicate the percentage change in outcome variables per 1-SD increment in plasma zinc and copper. Abbreviations: BMI, body mass index; CI, confidence interval; HDL, high-density lipoprotein cholesterol; LDL, low-density lipoprotein cholesterol.

**Table 3:** Associations of plasma Zn and Cu with subcutaneous and visceral fat (n = 534), and with liver signal intensity (n = 538).

MRI Traits as Outcome Variables	Zinc		Copper	
	Estimates (95% CI) ^a^	*p* Values	Estimates (95% CI) ^a^	*p* Values
Subcutaneous adipose tissue (dm^3^)				
Model B1	3.17 (−0.54; 7.03)	0.095	9.89 (6.02; 13.91)	<0.001
Model B2	3.77 (0.18; 7.48)	0.039	3.65 (−0.63; 8.11)	0.096
Model B3	3.60 (0.01; 7.31)	0.049	4.77 (0.44; 9.29)	0.031
Model B4	3.38 (−0.21; 7.10)	0.065	4.64 (0.31; 9.15)	0.036
Model B5	−0.37 (−2.22; 1.51)	0.697	0.46 (−1.77; 2.74)	0.688
Visceral adipose tissue (dm^3^)				
Model B1	7.60 (3.01; 12.40)	0.001	−12.07 (−15.74; −8.23)	<0.001
Model B2	5.78 (2.02; 9.68)	0.002	2.31 (−2.06; 6.88)	0.305
Model B3	5.69 (2.02; 9.50)	0.002	3.56 (−0.78; 8.08)	0.109
Model B4	5.73 (2.04; 9.56)	0.002	3.57 (−0.78; 8.10)	0.109
Model B5	2.40 (−0.07; 4.93)	0.057	−0.06 (−2.96; 2.91)	0.966
Liver signal intensity				
Model B1	6.32 (2.73; 10.04)	<0.001	−2.17 (−5.50; 1.29)	0.216
Model B2	5.83 (2.32; 9.45)	0.001	−3.70 (−7.53; 0.29)	0.069
Model B3	3.92 (1.57; 6.32)	0.001	−1.48 (−4.16; 1.29)	0.291
Model B4	3.84 (1.49; 6.25)	0.001	−1.49 (−4.18; 1.27)	0.287
Model B5	3.20 (0.90; 5.54)	0.006	−2.11 (−4.72; 0.56)	0.121

Model B1 was unadjusted. Model B2 was adjusted for age and sex. Model B3 was additionally adjusted for education, smoking habits, season, zinc supplementation (plasma zinc as exposure variable), lipid-lowering medication, prevalent fatty liver disease (for liver signal intensity as outcome variable), physical activity and alcohol consumption. Model B4 was additionally adjusted for total fat intake and intake of saturated fatty acids. Model B5 was additionally adjusted for BMI. Subcutaneous and visceral adipose tissue and liver signal intensity entered into the models as ln-transformed variables. ^a^ Regression coefficients indicate the % change in subcutaneous and visceral adipose tissue and liver signal intensity per 1-SD increment in plasma zinc and copper concentrations. Abbreviations: BMI, body mass index; CI, confidence interval; MRI, magnet resonance imaging; SD, standard deviation.

## Data Availability

Data described in the article, code book, and analytic code will be made available upon reasonable request.

## Supplementary Materials

The following supporting information on selection bias and sensitivity analyses can be downloaded at: https://www.mdpi.com/article/10.3390/nu15204460/s1, Table S1: Main characteristics of participants, separate for those individuals included and excluded in analyses in participants from the first follow-up examination (2010-2012) in the popgen-cohort from Northern Germany.; Table S2: Associations of plasma zinc and copper concentrations with anthropometric, metabolic and inflammatory traits in participants not taking zinc supplements (n = 784); Table S3: Associations of plasma Zn and Cu with subcutaneous and visceral fat (n = 534) and with liver signal intensity in participants not taking zinc supplements.

## Abbreviations

BMI	Body mass index
CI	Confidence interval
CRP	C-reactive protein
Cu	Copper
DEXA	Dual-energy X-ray absorptiometry
FFQ	Food frequency questionnaire
HDL	High-density lipoprotein cholesterol
LDL	Low-density lipoprotein cholesterol
LSI	Liver signal intensity
MRI	Magnet resonance imaging
NAFLD	Non-alcoholic fatty liver disease
NHANES	National Health and Nutrition Examination Survey
SAFs	Saturated fatty acids
SAT	Subcutaneous adipose tissue
TG	Triglycerides
SD	Standard deviation
VAT	Visceral adipose tissue
WC	Waist circumference
WHR	Waist-to-hip ratio
Zn	Zinc
