# Supplementary material for: Association of Plasma Zinc and Copper with Body Composition, Lipids and Inflammation in a Cross-Sectional General Population Sample from Germany
Source: Nutrients. 2023 Oct 20;15(20):4460. doi: 10.3390/nu15204460 (PMC10609917; doi:10.3390/nu15204460)
Supplement: Supplementary file 1 [file nutrients-15-04460-s001.zip › nutrients-2652535-supplementary.pdf]

nutrients

Supplementary Materials

Association of Plasma Zinc and Copper with Body Composition, Lipids and Inflammation in a Cross-Sectional General Population Sample from Germany

Cara Övermöhle [1,*], Gerald Rimbach [2], Sabina Waniek [1], Eike A. Strathmann [1], Tatjana Liedtke [1], Paula Stürmer [1], Marcus Both [3], Katharina S. Weber [1,†] and Wolfgang Lieb [1,†]

[1] Institute of Epidemiology, Kiel University, 24105 Kiel, Germany; katharina.weber@epi.uni-kiel.de (K.S.W.); wolfgang.lieb@epi.uni-kiel.de (W.L.)
[2] Institute of Human Nutrition and Food Science, Kiel University, 24118 Kiel, Germany
[3] Department of Diagnostic Radiology, University Hospital Schleswig-Holstein, 24105 Kiel, Germany
[*] Correspondence: cara.oevermoehle@epi.uni-kiel.de; Tel.: +49(0)431-500-30223
[†] These authors contributed equally to this work.

Table S1: Main characteristics of participants, separate for those individuals included and excluded in analyses in participants from the first follow-up examination (2010-2012) in the popgen-cohort from Northern Germany.

	Participants included in overall analysis (n=841)	Participants excluded from overall analysis (n=88)	*P*[*1]	Participants included in analysis of adipose tissue (n=534)	Participants excluded from analysis of adipose tissue (n=395)	*P*[*2]	Participants included in analysis of liver fat (n=538)	Participants excluded from analysis of liver fat (n=391)	*P*[*3]
n (% female) [a,d]	841 (42)	87 (52)	0.077	534 (41)	394 (45)	0.318	538 (42)	390 (43)	0.772
Plasma concentration of zinc (µg/L) [b]	695.0 ± 83.1	708.1 ± 132.2	0.187	699.6 ± 85.3	691.7 ± 93.4	0.180	698.6 ± 85.6	693 ± 93.3	0.347
Plasma concentration of copper (µg/L) [b]	1020.0 ± 903.4	1143.6 ± 991.7	<0.001	1014.6 ± 899.9	1052.0 ± 920.0	<0.001	1018.6 ± 906.0	1040.6 ± 914.9	0.001
Age (years) [b]	61 ± 12	56 ± 15	0.001	61 ± 12	60 ± 14	0.072	61 ± 12	59 ± 14	0.016
Body mass index (kg/m^2) [b]	27.2 ± 4.2	30.0 ± 8.7	<0.001	27.0 ± 3.9	28.1 ± 6.0	<0.001	27.0 ± 4.0	28.1 ± 5.9	<0.001
Current smokers (yes, (%)) [a,e]	108 (13)	22 (29)	<0.001	46 (9)	84 (22)	<0.001	49 (9)	81 (21)	<0.001
Alcohol intake (g/day) [c,f]	9.0 (3.2; 18.5)	7.6 (2.1; 18.8)	0.483	9.8 (4.0; 19.2)	7.6 (2.4; 17.2)	0.006	9.6 (3.5; 19.0)	8.1 (2.8; 18.0)	0.092
Physical activity (MET-hours/week) [c,f]	90.5 (59.0; 131.5)	71.8 (47.1; 103.3)	0.003	90.2 (59.4;132.1)	87.5 (56.3; 126.8)	0.169	91.4 (59.5; 131.7)	85.6 (55.3;125.8)	0.083
Education level (low [< 10 years], medium [10 years], high [≥ 11 years]) [a,g]	292 (35)<br>271 (32)<br>278 (33)	31 (37)<br>31 (37)<br>22 (26)	0.422	162 (30)<br>185 (35)<br>187 (35)	161 (41)<br>117 (30)<br>113 (29)	0.003	163 (30)<br>188 (35)<br>187 (35)	160 (41)<br>114 (29)<br>113 (29)	0.002

Values are [a] n (%) (categorical variables), [b] mean ± SD (continuous normally distributed variables); [c] median (IQR) (continuous skewed variables); data only available for [d] n=928, [e] n=917, [f] n=923, [g] n=925.

* *P* values based on chi-square test (categorical variables), Kruskal-Wallis test (continuous skewed variables) or general linear models (continuous normally distributed variables); [1] *P*-value for comparison between participants included vs. excluded from overall analysis; [2] *P*-values for comparison between participants included vs. excluded from adipose tissue analysis; [3] *P*-values for comparison between participants included vs. excluded from analysis of liver fat.

Abbreviations: CI, confidence interval; IQR, interquartile range; MET, metabolic equivalent of task; SD, standard deviation; T, tertile.

Table S2: Associations of plasma zinc and copper concentrations with anthropometric, metabolic and inflammatory traits in participants not taking zinc supplements (n=784).

Anthropometric, metabolic and inflammatory outcome variables	Zinc		Copper	
	Estimates (95% CI) [a]	*P values*	Estimates (95% CI) [a]	*P values*
Body mass index (kg/m^2)				
Model A1	1.56 (0.49; 2.64)	0.004	0.30 (-0.75; 1.36)	0.582
Model A2	1.35 (0.30; 2.42)	0.012	1.44 (0.20; 2.69)	0.023
Model A3	1.22 (0.18; 2.28)	0.021	1.44 (0.20; 2.69)	0.023
Model A4	1.22 (0.18; 2.28)	0.022	1.45 (0.21; 2.71)	0.022
Waist circumference (cm)				
Model A1	1.43 (0.48; 2.38)	0.003	-1.89 (-2.79; -0.99)	<0.001
Model A2	0.94 (0.11; 1.79)	0.027	1.16 (0.18; 2.16)	0.020
Model A3	0.86 (0.04; 1.70)	0.040	1.07 (0.09; 2.06)	0.033
Model A4	0.88 (0.05; 1.71)	0.038	1.10 (0.12; 2.09)	0.028
Waist-to-hip ratio				
Model A1	1.16 (0.52; 1.82)	<0.001	-2.48 (-3.08; -1.87)	<0.001
Model A2	0.69 (0.21; 1.16)	0.005	0.63 (0.07; 1.19)	0.027
Model A3	0.70 (0.23; 1.16)	0.003	0.40 (-0.15; 0.96)	0.153
Model A4	0.70 (0.24; 1.17)	0.003	0.42 (-0.14; 0.97)	0.141
Plasma triglyceride concentration (mg/dL)				
Model A1	3.36 (0.13; 6.68)	0.041	-1.51 (-4.56; 1.64)	0.343
Model A2	2.63 (-0.53; 5.88)	0.103	1.35 (-2.30; 5.13)	0.474
Model A3	1.81 (-1.21; 4.92)	0.242	-0.94 (-4.30; 2.55)	0.593
Model A4	1.73 (-1.29; 4.84)	0.265	-1.07 (-4.44; 2.42)	0.544
Plasma HDL concentration (mg/dL)				
Model A1	-2.01 (-3.82; -0.17)	0.032	7.32 (5.42; 9.25)	<0.001
Model A2	-1.37 (-3.01; 0.31)	0.110	1.32 (-0.66; 3.35)	0.193
Model A3	-0.44 (-2.00; 1.14)	0.583	2.86 (1.03; 4.73)	0.002
Model A4	-0.44 (-2.00; 1.15)	0.586	2.87 (1.03; 4.74)	0.002
Plasma LDL concentration (mg/dL)				
Model A1	3.30 (1.39; 5.24)	<0.001	3.11 (1.22; 5.04)	0.001
Model A2	3.21 (1.30; 5.15)	<0.001	3.60 (1.36; 5.89)	0.002
Model A3	3.75 (1.88; 5.66)	<0.001	2.72 (0.58; 4.90)	0.012
Model A4	3.74 (1.87; 5.64)	<0.001	2.66 (0.53; 4.84)	0.014
C-reactive protein (mg/L)				
Model A1	-6.70 (-12.84; -0.13)	0.046	38.68 (30.13; 47.79)	<0.001
Model A2	-6.91 (-12.99; -0.40)	0.038	46.98 (36.40; 58.38)	<0.001
Model A3	-8.85 (-14.25; -3.11)	0.003	37.48 (28.28; 47.35)	<0.001
Model A4	-8.91 (-14.32; -3.16)	0.003	37.47 (28.24; 47.36)	<0.001

Model A1 unadjusted. Model A2 adjusted for age and sex. Model A3 additionally adjusted for BMI (not for BMI, waist circumference and waist-to-hip ratio as exposure variable), education, smoking habits, season, lipid-lowering medication (for triglycerides, HDL and LDL as independent variables), fasting status (for triglycerides, HDL and LDL as independent variables) and physical activity and alcohol consumption. Model A4 additionally adjusted for total fat intake and intake of saturated fatty acids.

[a] Regression coefficients indicates the percentage change in outcome variables per 1-SD increment in plasma zinc and copper.

Abbreviations: BMI, body mass index; CI, confidence interval; HDL, high-density lipoprotein cholesterol; LDL, low-density lipoprotein cholesterol.

Table S3: Associations of plasma Zn and Cu with subcutaneous and visceral fat (n=534) and with liver signal intensity in participants not taking zinc supplements.

	Zinc		Copper	
**MRI traits as outcome variables**	Estimates (95% CI) [a]	*P values*	Estimates (95% CI) [a]	*P values*
Subcutaneous adipose tissue (dm^3)				
Model B1	3.58 (-0.26; 7.56)	0.068	9.43 (5.51; 13.49)	<0.001
Model B2	4.71 (0.98; 8.58)	0.013	3.22 (-1.14; 7.78)	0.150
Model B3	4.21 (0.50; 8.06)	0.026	4.23 (-0.19; 8.85)	0.061
Model B4	4.05 (0.32; 7.91)	0.033	4.14 (-0.29; 8.76)	0.067
Model B5	-0.33 (-2.28; 1.67)	0.746	0.67 (-1.66; 3.07)	0.575
Visceral adipose tissue (dm^3)				
Model B1	10.29 (5.47; 15.32)	<0.001	-12.46 (-16.17; -8.59)	<0.001
Model B2	7.31 (3.36; 11.41)	<0.001	1.52 (-2.96; 6.21)	0.512
Model B3	6.77 (2.94; 10.75)	<0.001	2.62 (-1.81; 7.25)	0.250
Model B4	6.87 (3.01; 10.87)	<0.001	2.65 (-1.79; 7.30)	0.246
Model B5	2.98 (0.37; 5.66)	0.025	-0.35 (-3.35; 2.75)	0.823
Liver signal intensity				
Model B1	7.16 (3.34; 11.11)	<0.001	-2.32 (-5.80; 1.29)	0.205
Model B2	6.60 (2.87; 10.46)	<0.001	-4.21 (-8.21; -0.04)	0.048
Model B3	4.71 (2.24; 7.24)	<0.001	-1.12 (-3.94; 1.79)	0.447
Model B4	4.64 (2.16; 7.18)	<0.001	-1.14 (-3.96; 1.77)	0.440
Model B5	3.82 (1.42; 6.29)	0.002	-1.74 (-4.47; 1.06)	0.219

Model B1 unadjusted. Model B2 adjusted for age and sex. Model B3 additionally adjusted for education, smoking habits, season, lipid-lowering medication, prevalent fatty liver disease (for liver signal intensity as outcome variable), physical activity and alcohol consumption. Model B4 additionally adjusted for total fat intake and intake of saturated fatty acids. Model B5 additionally adjusted for BMI

Subcutaneous and visceral adipose tissue and liver signal intensity entered into the models as ln-transformed variables.

[a] Regression coefficients indicates the %-change in subcutaneous and visceral adipose tissue and liver signal intensity per 1-SD increment in plasma zinc and copper concentrations

Abbreviations: BMI, body mass index; CI, confidence interval; MRI, magnet resonance imaging; SD, standard deviation.
